# Inquiry-based training improves teaching effectiveness of biology teaching assistants 

**DOI:** 10.1371/journal.pone.0078540

**Published:** 2013-10-11

**Authors:** P. William Hughes, Michelle R. Ellefson

**Affiliations:** 1 Department of Biology, Carleton University, Ottawa, Ontario, Canada; 2 Faculty of Education, University of Cambridge, Cambridge, United Kingdom; University of Minho, Portugal

## Abstract

Graduate teaching assistants (GTAs) are used extensively as undergraduate science lab instructors at universities, yet they often have having minimal instructional training and little is known about effective training methods. This blind randomized control trial study assessed the impact of two training regimens on GTA teaching effectiveness. GTAs teaching undergraduate biology labs (n = 52) completed five hours of training in either inquiry-based learning pedagogy or general instructional “best practices”. GTA teaching effectiveness was evaluated using: (1) a nine-factor student evaluation of educational quality; (2) a six-factor questionnaire for student learning; and (3) course grades. Ratings from both GTAs and undergraduates indicated that indicated that the inquiry-based learning pedagogy training has a positive effect on GTA teaching effectiveness.

## Introduction

Graduate teaching assistant (GTA)-run introductory science courses are the norm at higher education institutions in North America, Australia and New Zealand, and are becoming more prevalent elsewhere [1]. GTAs are part-time employees (often research students) hired to lead lab sessions, grade papers, and provide assistance to course instructors, and account for many of the contact hours undergraduates have with the department. GTAs have a powerful influence on undergraduate student learning, but not without cost [2,3]. Graduate students gain income and teaching experience, and science departments gain a pool of inexpensive laborers versed in discipline-specific content, but because GTA teaching can be inconsistent, and students find it difficult to learn from inexperienced teachers, ensuring that undergraduates receive high-quality teaching from GTAs is one of the main teaching issues facing science departments [4–7].

One solution includes general teaching training for GTAs, but the effectiveness of this training is mixed [2–4,8,9]. The training content is not standardized, as it is for schoolteachers, and attendance is not compulsory. A 1997 survey of 153 biology departments in the United States indicated that 49% of GTA programs required no formal training whatsoever [10]. For those programs that do require formalized GTA training, the form that training takes can vary widely, from university-wide mass orientation workshops to subject-based instruction from instructors, supervisors or peers [4]. Content might be as important as format – a 2004 study cited the lack of adequate preparation for facilitating open inquiry labs as a major difficulty both for undergraduate students and the GTAs themselves [5]. 

Well-constructed science lab activities are powerful learning tools; through guided inquiry, undergraduates gain first-person experience of scientific principles and phenomena learned in lectures, and learn to employ experimental methods to solve discrete problems [11]. As such, training that exposes GTAs to the inquiry process may improve teaching effectiveness. Common misperceptions about undergraduate labs include: (a) that the purpose of lab activity is to recapitulate content learned through lectures or from readings; or (b) that the purpose is to familiarize students with cutting-edge experimental techniques. Such objections ignore what is unique about the lab experience – the opportunity to practice applying the scientific method. Many introductory labs deliberately use older, highly predictable experimental setups to teach students basic scientific inquiry skills. 

Inquiry-based learning is a pedagogical approach rooted in constructivist learning theories that advocates teaching cognitive skills through open-ended exploration by the learner ([12,13]). Inquiry activities commonly include designing protocols, developing procedures and proposing unified explanations for experimental or anecdotal phenomena. Structured-inquiry activities, which are commonly used as undergraduate lab exercises, typically ask students to propose specific explanations, rooted in relevant course content, for data gathered from experimental trials [14,15]. Teaching “facts”, “knowledge” or “content” is only the secondary objective of such activities: the main goal is to develop the reasoning skills that are required to plan, execute and interpret scientific experiments.

Successful inquiry-oriented initiatives such as POGIL (Process Oriented Guided Inquiry Learning) emphasize the coordination of content and methodological skills during science learning activities [16–18], but implementing large-scale reorganization of teaching materials and curricular content calls for substantial investment in time and money, and may be beyond the capability of many course instructors. Moreover, reorienting major introductory course lecture content toward inquiry learning is a considerable effort, and requires departmental consensus to implement effectively. The current study investigated whether it is possible to increase learning effectiveness in an inquiry-based activity without taking on large-scale curricular redesign.

Previous studies have suggested that training GTAs on the use of inquiry-based methods will improve GTA teaching effectiveness in undergraduate labs [19,20]. Although few empirical studies have identified which kinds of GTA training content actually improve general lab GTA teaching effectiveness, and none have specifically focused on inquiry-based methods, some authors have used correlational data or made general recommendations regarding GTA teaching [7,13,21]. Inquiry-based methods may be of particular value to GTAs teaching introductory labs, since labs are designed to teach scientific enquiry as well as course content [22–24].

Here, our aim was to test whether grounding GTA training in inquiry-based learning theory would measurably improve teaching effectiveness in undergraduate biology labs, as assessed by undergraduate and GTA responses to online questionnaires, as well as standardized student grades. We predict that explaining inquiry-based methods would improve lab GTA teaching performance, although it might also be reasonable to argue that the limited time allotted might be better spent if allocated to practical teaching activities rather than theoretical ones because GTA training is extremely brief (typically less than five hours per year). In order to test this hypothesis, we conducted a semi-randomized control trial to compare two training regimens for GTAs of introductory biology labs: a control regimen that is taught various “best practices” associated with lab teaching; and an experimental regimen that explained inquiry-oriented pedagogy to GTAs.

## Methods

### i) Participants

This study took place during the first semester of the academic year at a large research-intensive North American university with about 25,000 students. Of a total number of 126 GTAs, 54 GTAs volunteered to take part in the study and met the study inclusion criteria. Each of these GTAs was employed as a GTA in a single lab section of an introductory biology course in the Fall 2012 semester, and was a graduate student registered in an MSc or PhD programme in the Department of Biology during the same semester. An online pre-assignment questionnaire recorded experience and program data for each GTA two weeks before the onset of the training. On the basis of the data collected in this questionnaire, participants were divided into two groups based on academic program (Masters or PhD). GTAs were allocated to either a “best practice” (Control) or inquiry-based learning pedagogy (Inquiry) training group. This allocation was semi-random; the proportion of GTAs in either academic program was held constant between training groups to balance the experimental design and avoid a confounding factor of academic program, but was otherwise random. Two participants from the control group discontinued the study after the first training session; 52 GTAs finished the training sessions (24 control and 28 inquiry GTAs) and all of these 52 participants completed an online survey conducted at the end of the semester.

GTAs were blind to their training group assignment and were not given specific details about the experimental design. Details about their lab section were collected during the training group workshops and from the online survey. This information allowed for pre-training responses to be matched with later survey responses as well as matching undergraduate students to their lab section GTA while protecting participant confidentiality.

Undergraduate students were recruited to rate their GTAs through advertisement posters, email invitations, and social media announcements. Undergraduates were told that GTAs were part of a training experiment, and were part of more than one GTA training group, but were not told: (a) what the training groups were, or (b) which training group their GTA had been allocated to. A total of 602 undergraduates provided responses, with 352 (58.5%) completing the entire online survey. Approximately 1,250 undergraduate students were enrolled in undergraduate biology during this semester. All GTA lab groups were represented by at least one undergraduate.

At the end of the academic semester, all 16 course professors were contacted by email and notified of the nature of the training experiment (i.e. that GTA training was being manipulated in a controlled experiment – although the training groups, training regimens and the GTAs involved were not revealed), then asked to provide average course grades. Average course marks for 49 of the 52 sections taught by GTAs taking part in the experiment were collected.

### ii) Training Workshops

The general format of the training for both groups was the same and the same instructor taught all sessions. Each training group attended a pair of workshops; each workshop lasted 2.5 hours for a total of 5 hours of training. The two workshops were held one week apart. The first workshop was oriented toward teaching and the second was oriented toward grading. All workshops involved a mix of PowerPoint® slides, group discussions, peer-led problem-solving sessions and question-and-answer sessions. Both training groups included the same core topics. The first session included information on: lecturing, moderating group discussions, facilitating lab activities, diagnosing problems in student understanding, giving effective prelab talks, dealing with student questions, and encouraging/motivating students in the lab. The second session included material related to marking: grading lab reports, providing useful feedback, rubric design, and speed grading tips.

Because our aim was to investigate whether inquiry pedagogy training improved GTA teaching effectiveness, the training groups mainly differed in terms of whether inquiry-based learning was explained as a theory of learning to be used in the science lab. Content for both GTA training groups was drawn from previous recommendations [9,25,26] and was common between the two training groups. 

The areas of teaching focus for the control (“best practices”) training group seminars were: (1) to identify effective and efficient strategies for GTAs to teach content knowledge to undergraduates during lab activities; and (2) to ensure that lab activity assessments were marked fairly. In general, the material taught to this group was designed to reflect the status quo GTA training curriculum at North American universities, and so “best practices” identified in GTA training materials or educational studies were provided to GTAs [26–28]. The training group focused on specific teaching practices of the GTAs themselves rather than explaining how to teach scientific enquiry; because many of the “best practices” for GTAs related to the teaching of content knowledge (i.e. “tips for teaching undergraduate students to use a light microscope”) rather than the scientific method, this training group did not explicitly teach GTAs how to teach higher-order cognitive skills. A full list of the activities that took place in the two control training group seminars (teaching and assessment) is given in Tables 1 and 2.

**Table 1:** Teaching Seminar Activity Chart for Control GTA Training Group.

**Item**	**Time Allocated**	**Activity**	**Desired Learning Outcome**	**Teaching Method**
1	30 min	GTA General Orientation	- understanding the role of GTAs	Instructor lecture (Powerpoint)
2	15 min	Brainstorming Activity: Laboratory Learning Outcomes	- identification of determinants of lab learning success	Student-led small groups, answers shared with class and discussed
3	45 min	Lab teaching best practices	- understanding of evidence-based methods for teaching effectively	Instructor lecture (Powerpoint)
			- understanding specific teaching skills, learning styles and learning outcomes	
4	30 min	Mini-microteaching Activity - Sample Lab Scenarios (light microscope)	- understanding of how to apply best practice teaching techniques in lab activity situations	Microteaching, large group activity
5	15 min	Troubleshooting inquiry-learning activities	- how to handle unexpected problems in lab activities	Instructor lecture (Powerpoint)
6	15 min	Questions	- clear up unresolved questions	Student-led question and answer

**Table 2:** Assessment Seminar Activity Chart for Control GTA Training Group.

**Item**	**Time Allocated**	**Activity**	**Desired Learning Outcome**	**Teaching Method**
1	30 min	History and Purpose of Assessment	- understanding of role of assessment in the learning process	Lecture (Powerpoint)
2	15 min	Brainstorming Activity: Laboratory Assessment Methods	- GTA identification of purpose of assessment in labs; criteria determining successful assessment methods for labs	Student-led small groups, answers shared with class and discussed
3	30 min	Grading Best Practices	- understanding of evidence-based methods for grading effectively	Instructor Lecture (Powerpoint)
			- understanding specific grading requirements for in-lab and lab report activities	
4	45 min	Small Group Rubric Design	- how rubrics are interpreted	Student-led small groups, answers shared with class and discussed
			- understanding rubric inclusion criteria	
			- understanding how feedback is different in-lab versus on lab reports	
5	15 min	How to Grade Equitably and Quickly	- how to maintain grading consistency	Instructor Lecture (Powerpoint)
			- how to grade lab feedback quickly	
6	15 min	Questions	- clear up unresolved questions	Student-led question and answer

For the inquiry pedagogy training group, teaching and grading were presented from a constructivist perspective: learning objectives were explained using the revised Bloom’s taxonomy, scaffolding and inquiry facilitation were explained as teaching methods that help students learn methodological skills, and giving feedback was oriented toward encouraging further inquiry [13,20,21,29–33]. The areas of teaching focus for the inquiry pedagogy training group seminars were: (1) to teach GTAs how inquiry-based practices teach students to reason independently and apply the scientific method; (2) to teach GTAs how to facilitate structured inquiry and open-ended learning as lab activities; and (3) to teach GTAs how to assess inquiry. Instead of teaching GTAs to teach undergraduates how to use a light microscope, GTAs were told instead how to facilitate and evaluate student-centered inquiry with respect to using a light microscope to ask scientific questions. A full list of the activities that took place in the two inquiry-based learning pedagogy training group seminars (teaching and assessment) is given in Tables 3 and 4.

**Table 3:** Teaching Seminar Activity Chart for Inquiry-based Learning Pedagogy GTA Training Group.

**Item**	**Time Allocated**	**Activity**	**Desired Learning Outcome**	**Teaching Method**
1	30 min	GTA General Orientation	- understanding the role of GTAs	Instructor lecture (Powerpoint)
2	15 min	Brainstorming Activity: Laboratory Learning Outcomes	- identification of determinants of lab learning success	Student-led small groups, answers shared with class and discussed
3	45 min	Introduction to Inquiry-based Learning	- understanding of the Revised Bloom’s Taxonomy of learning	Instructor lecture (Powerpoint)
			- how scientific enquiry is taught using inquiry	
			- understanding of facilitation as a teaching method	
4	30 min	Facilitation Situation Activity - Sample Lab Scenarios	- understanding of how to apply facilitation techniques in teaching situations	Large group activity
5	15 min	Troubleshooting inquiry-learning activities	- how to handle unexpected problems in inquiry-based lab activities	Instructor lecture (Powerpoint)
6	15 min	Questions	- clear up unresolved questions	Student-led question and answer session

**Table 4:** Assessment Seminar Activity Chart for Inquiry-based Learning Pedagogy GTA Training Group.

**Item**	**Time Allocated**	**Activity**	**Desired Learning Outcome**	**Teaching Method**
1	30 min	History and Purpose of Assessment	- understanding the role of assessment in the learning process	Instructor lecture (Powerpoint)
2	15 min	Brainstorming Activity: Laboratory assessment Methods	- identification of: (1) purpose of assessment in labs; (2) criteria determining successful assessment methods for labs	Student-led small groups, answers shared with class and discussed
3	30 min	How to Assess Scientific Inquiry	- understanding of the distinction between lower- and higher-order cognitive skills	Instructor lecture (Powerpoint)
			- understanding how to use assessment tolls to give feedback on cognitive skill development	
4	15 min	Assessment Rubric Examples	- how rubrics are interpreted	Student-led question and answer
5	45 min	Small Group Rubric Design	- how rubrics are interpreted	Student-led small groups, answers shared with class and discussed
			- understanding rubric inclusion criteria	
			- understanding how feedback can assess different cognitive skills	
6	15 min	Questions	- clear up unresolved questions	Student-led question and answer

The common two-seminar format meant that both training groups used the same peer-led learning activities. For example, GTAs of both training groups paired up to spend time practicing answering common student questions – but these took different forms based on the learning goals of the respective training group. For example, GTAs in the control training group practiced answering questions about lab protocol by answering common questions about the relevant section of the lab manual. GTAs in the inquiry pedagogy training group practiced answering questions about lab protocol by learning to design questions to help students figure out the next steps in the protocol for themselves. Extensive efforts were made to ensure the consistency and reliability of the workshops for both training groups. GTAs in both training groups were instructed not to discuss the content of their GTA training sessions with one another or with undergraduates. 

### iii) Measures

We assessed teaching effectiveness for the GTAs in the two training groups using three measures: (a) a 32-item, nine-factor student evaluation of educational quality (SEEQ) [34–36]; (b) a 6-item cognitive learning evaluation (CLE) questionnaire; and (c) standardized mean student grade. 

#### a) Student Evaluation of Educational Quality

The Student Evaluation of Educational Quality (SEEQ) inventory is a nine-factor, 32-item validated survey instrument that has been widely used to evaluate undergraduate instruction at North American universities. The original SEEQ inventory [34] was written to appraise course instructors, but for this study we modified it to evaluate GTAs by simple modifications of the text (see Table 5). Nine identifiable dimensions of teaching quality can be evaluated using SEEQ: (1) learning/academic value; (2) instructor enthusiasm; (3) organization; (4) group interaction; (5) individual rapport; (6) breadth of coverage; (7) examination and grading; (8) assignments; and (9) overall instructional ability.

**Table 5:** Student Evaluation of Educational Quality Factor Descriptions and Example Items.

**SEEQ Factor**	**Items**	**Description**	**Sample Item**
(1) Learning/ Academic Value	4	Refers to the feeling of achievement and academic success that students obtain from participation in a course. High instructor ratings indicate that the instructor is successfully imparting useful information to students, and helping them feel that what they have learned is worthwhile and challenging.	“Through my GTA, I have learned and understood the subject materials in this course”
(2) Instructor Enthusiasm	4	Refers to the instructor’s ability to created attentiveness and interest in the educational material on behalf of the student. High instructor ratings indicate that the instructor is creating engagement through dynamic presentation, and relating course material in a way that evokes interest.	“My GTA was energized and dynamic in conducting the course”
(3) Organization	4	Refers to the structure and transparency of the instructor’s explanation of subject matter. High instructor ratings indicate that the instructor is relating information clearly and precisely, in a way that is easy for students to understand.	“GTA explanations were clear”
(4) Group Interaction	4	Refers to the ability to foster academically useful social interactions within the classroom. High instructor ratings indicate that the instructor is encouraging group work in a positive way, and is motivating students to share knowledge effectively.	“Students were invited to express their own ideas and/or question the GTA”
(5) Individual Rapport	4	Refers to the capacity to engage personally with individual learners and provide academically significant help and encouragement. High instructor ratings indicate that the instructor is able to relate to students on a personal level and provide meaningful guidance.	“My GTA had a genuine interest in individual students”
(6) Breadth of Coverage	4	Refers to the ability to explain and compare alternative ideas, theories and techniques in a way that highlights essential features. High instructor ratings indicate that the instructor is able to relate knowledge to students effectively through contrasting specific ideas.	“My GTA contrasted the implications of various theories”
(7) Examination and Grading	3	Refers to the ability to provide fair and useful evaluative feedback. High instructor ratings indicate that the instructor equitably assesses student work and provides meaningful correction to students.	“My GTA’s feedback on examinations/graded materials was valuable”
(8) Assignments	2	Refers to the ability to create or use assignments to relate material to students. High instructor ratings indicate that the instructor is capable of designing or implementing assessments in such a way that new subject matter is taught or that errors are corrected.	“Readings/texts/references suggested by my GTA were valuable”
(9) Overall Instructional Ability	2	A general evaluation of teaching effectiveness.	“Overall, my GTA was a good teacher”

Note: sample items shown refer to the undergraduate survey; GTA survey contained the same items, but replaced “my GTA” with “I” (e.g., “Overall, I am a good teacher”). Items were rated on a five-point Likert scale (strongly disagree, disagree, neutral, agree, strongly agree) with a “NA/Don’t Know” null response included.

We used the first eight factors from Marsh’s original SEEQ inventory to create two SEEQ instruments (available from the original papers [28,30]). We changed Factor 9 from its original (1982) version. Marsh’s 1982 version of the SEEQ inventory did not use “overall instructional ability” but rather “course difficulty” as the ninth SEEQ factor [34,36]. GTAs do not have control over course difficulty because they do not design the course. As such, we opted to use a newer version of SEEQ developed by the University of Saskatchewan, which includes “overall instructional ability” as factor 9. After these modifications, two SEEQ instruments were created: one was used by GTAs for self-evaluation, and the other by undergraduates to rate their GTA (see Table 5 for examples) 

SEEQ responses were given using a five-point Likert scale and scored as: “strongly disagree” = 1; “disagree” = 2; “neutral”= 3; “agree”= 4; and “strongly agree”= 5. When participants selected “not applicable”, then that question was given a null value and excluded from further analyses. 

#### b) Cognitive Learning Evaluation

The cognitive learning evaluation (CLE) instrument is a short six-factor questionnaire evaluating individual learning outcomes derived from Bloom’s revised taxonomy of learning [37]. The six factors assessed were: (1) knowledge; (2) comprehension; (3) problem-solving; (4) conceptual-analytic; (5) planning; and (6) evaluation (Table 6). Factors occur in a hierarchical order of increasing learning depth and difficulty of instruction that assumed that higher factors represent more in-depth learning. Each CLE factor was assessed by a single item. Responses were collected using a five-point Likert scale and scored as: “strongly disagree”= 1; “disagree” = 2; “neutral”= 3; “agree”= 4; and “strongly agree”= 5. When participants selected “not applicable”, then that question was given a null value and excluded from further analyses. 

**Table 6:** Cognitive Learning Evaluation Factor Descriptions and Example Items.

**CLE Factor**	**Abstract Example**	**Concrete Example**	**Item**
(1) Knowledge	Recalling information	Learning exact molecular weights from the periodic table	“My GTA helped me learn knowledge skills.”
(2) Comprehension	Comparing or contrasting two ideas	Learning to restate a word problem using equations	“My GTA helped me learn comprehension skills.”
(3) Application/Problem-solving	Applying knowledge to find a solution to a specific question	Learning to select the appropriate statistical test for an analysis	“My GTA helped me learn problem-solving skills.”
(4) Conceptual Analysis	Determining causes and identifying relationships	Learning to troubleshoot a lab protocol	“My GTA helped me learn conceptual-analytic skills.”
(5) Planning	Creating a strategy by using ideas in a new way	Learning to design a new lab protocol using first principles	“My GTA helped me learn planning skills.”
(6) Evaluation	Using critical reasoning to make specific judgments about ideas	Learning to identify the most relevant theoretical approach to design a set of experiments	“My GTA helped me learn evaluation skills.”

Note: sample items shown refer to undergraduate survey; GTA survey contained the same items, but replaced “my GTA” with “I” and “me” with “undergraduate students” (e.g., “I helped undergraduate students learn problem-solving skills”). Items were rated on a five-point Likert scale (strongly disagree, disagree, neutral, agree, strongly agree) with a “NA/Don’t Know” null response included.

#### c) Grades

Undergraduates self-reported their final grades for the course. Final grades reflected contributions from both lab and lecture components, although GTAs graded only lab assignments. Grading frameworks differed amongst course professors, with the lab component accounting for 25-50% of the final grade. Final exams were the largest single other contribution to final grades, ranging 30-75% of the final grade and usually included multiple-choice, objectively graded questions (i.e., by Scantron). The objective nature of the final course grades should mitigate the likelihood that GTA grading standards differed between training groups (influenced by a Hawthorne effect), or that common examinations and syllabi were not used by course professors.

Reported final grades fell on the 12-point GPA (grade point average) scale used by the university where this study took place. Using this scale, student work is graded by percentile score (0-100%), which is linked to a corresponding letter and GPA grade (e.g., 90-100% = A+ or 12.0; GPA 73-76.9% = B or 8.0 GPA; 60-62.9% = C- or 4.0 GPA; 53-56.9% = D or 2.0 GPA). Global course mean GPA values were collected from course professors, and grades were standardized by subtracting course mean GPA scores (calculated by averaging undergraduate self-reported grades per course) from individual GTA lab group GPA scores. The resulting standardized grades ranged from 3.7 to -3.1, and represented how much better (i.e., positive values) or worse (i.e., negative values) a given GTA lab group mean grade was than the average grade for any group in that course (in terms of GPA). Standardized grades were used instead of raw grades to account for the variation between courses, professors and lab material. 

### iv) Procedures

GTAs completed the pre-assignment questionnaires during the first week of a 13-week university semester. Training sessions were held on the third and fifth weeks of that same semester. 

The SEEQ, CLE, and other demographic measures were collected from GTAs and undergraduates using a 116-item online survey hosted by Qualtrics Inc. The surveys for GTAs and undergraduate students were nearly identical, with only minor modifications relevant to the their roles. (e.g., references to “me (the GTA)” for the GTA survey were “my GTA” in the undergraduate survey). GTAs completed the surveys near the end of the semester. Approximately 7 weeks elapsed between the first GTA training session and the completion of the survey. Undergraduates were surveyed during a 45-day period beginning on the last day of exams for that semester. Data from course professors were collected during this same 45-day period.

### v) Analyses

A pretest logistic regression of program distribution indicated that there were no significant differences in the ratio of Masters to PhD students between training groups (R^2^= .0003, χ^2^_1,1_ = 0.02, p = .895). A similar pretest one-way ANOVA showed no significant difference in GTA experience (in years of teaching) between the two training groups (*F*(1,50) = 0.01, *p*= .933). Shapiro-Wilks tests indicated that all of the dependent variables were normally distributed.

For the SEEQ and CLE questionnaires, we ran multivariate analyses because the responses were composed of multiple non-independent subscales. Responses for these instruments were analyzed using a 2x2 between-subjects MANCOVA, and follow-up ANCOVA tests were performed where the multivariate response was significantly predicted by training group (similar to the analyses suggested in other studies [38,39]. For follow-up tests, Type I error was controlled for using a Bonferroni-corrected alpha value (e.g., the SEEQ inventory had 9 factors, so we used α = .0056). We ran separate MANCOVAs for GTA self-evaluation (*n* = 52 for both SEEQ and CLE) and for undergraduate evaluation (*n* = 47 for SEEQ, *n* = 50 for CLE). Two independent variables were included in our MANCOVAs: training group (control or inquiry) and GTA academic program (Masters or PhD), while previous GTA experience (in years of teaching) was included as a covariate. The dependent variables were all of the nine SEEQ and six CLE factors. GTA self-responses are analyzed as raw ratings. Undergraduate responses were pooled for each GTA lab section and analyzed as a mean rating for each GTA. We assessed significance of training group as a multivariate effect using the Pillai’s Trace statistic, since Pillai’s Trace is the multivariate measure which is both: (1) most robust to multivariate non-normality; and (2) conservative when the sample size is small (<100) [40].

We ran each MANCOVA without the covariate (i.e., as a MANOVA) to see if the significance levels of any multivariate or univariate effects depended on its inclusion. These analyses indicated that this was not the case, and as such, we therefore present only the MANCOVA results here.

To analyze grade data, we used a two-factor ANCOVA (*n* = 49) to examine standardized grade scores, again with training group (control or inquiry) and GTA academic program (Masters or PhD) as independent variables and GTA experience (in years of teaching) as a covariate. Again, the analysis was re-run without the covariate (i.e., as an ANOVA) and the overall findings were consistent with the ANCOVA results. As such, only the ANCOVA results are reported here.

All statistical analyses were performed using SPSS 20. Effect sizes are reported using partial *η* ^2^ for multivariate tests and *r* for univariate tests. Partial *η* ^2^ indicates the estimated amount of the total variation in the response due to variation in an individual predictor [41]. 

### vi) Research Ethics

This study was examined and approved by the Carleton University Research Ethics Board (Project Number 13-0563). Written informed consent was obtained from all human subjects – both GTA participants and undergraduate survey respondents – involved in this study. Subjects were specifically informed that: (1) they were under no obligation to continue their participation in the research project; (2) that there were no known risks associated with participation; and (3) no identifying information would be collected, responses would be kept anonymous, and results would be reported only in aggregate.

## Results

### a) Student Evaluation of Educational Quality Results

There was not a significant effect of the covariate - GTA experience (in years) - on the undergraduate responses to the SEEQ questionnaire (see Table 3 for a list of SEEQ Factors) (*Pillai’s trace* = 0.18, *F*(9,34) = 0.81, *p* = .61, *partial η* ^2^ = 0.18, observed power = .33). There was a significant multivariate main effect for training group, (*Pillai’s trace* = 0.51, *F*(9,34) = 3.93, *p* = .002, *partial η* ^2^ = 0.51, observed power = .98; Table 7). However, there was neither a significant effect of GTA academic program (*Pillai’s trace* = 0.16, *F*(9,34) = 0.70, *p* = .71, *partial η* ^2^ = 0.16, observed power = .28) nor a significant interaction between training group and GTA academic program (*Pillai’s trace* = 0.29, *F*(9,34) = 1.57, *p* = .16, *partial η* ^2^ = 0.29, observed power = .63). 

**Table 7:** MANCOVA test results for undergraduate Student Evaluation of Educational Quality evaluation ratings (*n* = 47, α = .05).

**Effect / Interaction**	***Pillai's Trace***	***F***	***df***	***p***	***partial η^2^***	**power**
GTA Experience (in years teaching)	0.18	0.81	9, 34	.609	0.18	.33
Training Group	0.51	3.93	9, 34	.002*	0.51	.98
GTA Academic Program	0.16	0.70	9, 34	.705	0.16	.28
Training Group × GTA Academic Program	0.29	1.57	9, 34	.163	0.29	.63

* *p*<.05, two-tailed.

**Figure 1:**
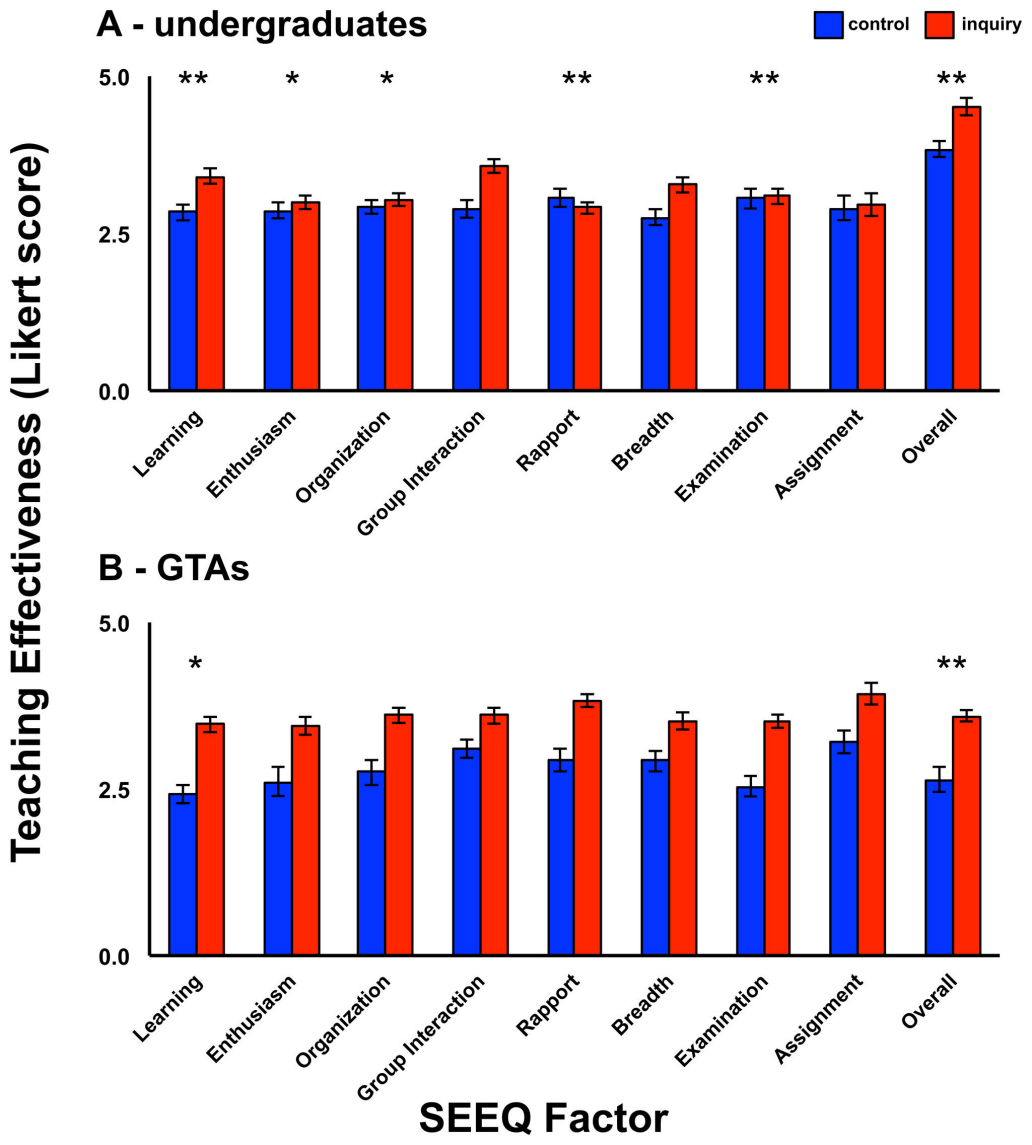
Mean undergraduate (A) and graduate teaching assistant (B) evaluations of GTA teaching effectiveness by Student Evaluation of Educational Quality (SEEQ) inventory factor for the “best practices” (control) and inquiry-based learning pedagogy (inquiry) training groups (with standard error bars). Paired columns with stars are significantly different (Bonferroni-corrected significance levels: *p<0.0083, **p<0.001).

For the undergraduate responses, there were significant univariate main effects for training group (using a Bonferroni-corrected α = .0056) on six of the nine SEEQ factors: learning (SEEQ Factor 1), *F*(1,42) = 26.47, *p* < .0005, *r* = .62; enthusiasm (SEEQ Factor 2), *F*(1,42) = 10.30, *p* < .003, *r* = .44; organization (SEEQ Factor 3), *F*(1,42) = 12.09, *p* < .001, *r* = .47; rapport (SEEQ Factor 5), *F*(1,42) = 14.47, *p* < .0005, *r* = .51; assignments (SEEQ Factor 7), *F*(1,42) = 25.79, *p* < .0005, *r* = .62; and overall instructional ability (SEEQ Factor 9), *F*(1,42) = 18.64, *p* < .0005, *r* = .55 (Figure 1, Table 8). A follow-up MANOVA revealed no difference in the significance level of any multivariate or univariate effects.

**Table 8:** ANCOVA test results for undergraduate Student Evaluation of Educational Quality evaluation ratings (*n* = 47, α = .0056).

**SEEQ Factor**	***F***	***df***	***p***	***r***	**Training Group**	**Mean**	**SE**
(1) Learning	26.47	1, 42	<.001**	.62	control	2.83	0.13
					inquiry	3.41	0.12
(2) Instructor Enthusiasm	10.30	1, 42	.003*	.44	control	2.86	0.13
					inquiry	3.00	0.11
(3) Organization	12.09	1, 42	.001*	.47	control	2.92	0.12
					inquiry	3.04	0.11
(4) Group Interaction	7.45	1, 42	.009	.39	control	2.89	0.14
					inquiry	3.57	0.12
(5) Individual Rapport	14.47	1, 42	<.001**	.51	control	3.06	0.15
					inquiry	2.91	0.10
(6) Breadth of Coverage	6.81	1, 42	.013	.37	control	2.76	0.13
					inquiry	3.28	0.13
(7) Examination	25.79	1, 42	<.001**	.62	control	3.06	0.16
					inquiry	3.10	0.13
(8) Assignments	5.86	1, 42	.020	.35	control	2.90	0.19
					inquiry	2.96	0.19
(9) Overall Instructional Ability	18.64	1, 42	<.001**	.55	control	3.84	0.14
					inquiry	4.52	0.14

* *p*<.0056, two-tailed. ** *p* <.0001, two-tailed.

There was not a significant effect of GTA experience (in years) on the GTA responses to the SEEQ questions (*Pillai’s trace* = 0.22, *F*(9,39) = 1.22, *p* = .31, *partial η* ^2^ = 0.22, observed power = .51). There was a significant multivariate main effect for training group, (*Pillai’s trace* = 0.38, *F*(9,39) = 1.68, *p* = .017, *partial η* ^2^ = 0.38, observed power = .89; Table 9). However, there was neither a significant main effect of GTA academic program (*Pillai’s trace* = 0.15, *F*(9,39) = 0.74, *p* = .67, *partial η* ^2^ = 0.15, observed power = .31) nor a significant interaction between training group and GTA academic program (*Pillai’s trace* = 0.22, *F*(9,39) = 1.19, *p* = .33, *partial η* ^2^ = 0.22, observed power = .50). 

**Table 9:** MANCOVA test results for GTA Student Evaluation of Educational Quality ratings (*n* = 52, α = .05).

**Effect / Interaction**	***Pillai's Trace***	***F***	***df***	***p***	***partial η^2^***	**power**
GTA Experience (in years teaching)	0.22	1.22	9, 39	.312	0.22	.51
Training Group	0.38	2.65	9, 39	.017*	0.38	.89
GTA Academic Program	0.15	0.74	9, 39	.670	0.15	.31
Training Group × GTA Academic Program	0.22	1.19	9, 39	.328	0.22	.50

* *p*<.05, two-tailed.

For the GTA responses, there were significant univariate main effects for training group (using a Bonferroni-corrected α = .0056) for three of the nine SEEQ factors: learning (SEEQ Factor 1), *F*(1,47) = 11.86, *p* < .001, *r* = .45; group interaction (SEEQ Factor 4), *F*(1,47) = 11.86, *p* < .001, *r* = .48; and overall instructional ability (SEEQ Factor 9), *F*(1,47) = 15.34, *p* < .0005, *r* = .50 (Figure 1, Table 10). A follow-up MANOVA revealed no difference in the significance level of any multivariate or univariate effects. 

**Table 10:** ANCOVA test results for GTA Student Evaluation of Educational Quality ratings (*n* = 52, α = .0056).

**SEEQ Factor**	***F***	***df***	***p***	***r***	**Training Group**	**Mean**	**SE**
(1) Learning	11.86	1, 47	.001*	.45	control	2.42	0.14
					inquiry	3.47	0.11
(2) Instructor Enthusiasm	1.98	1, 47	.17	.20	control	2.61	0.21
					inquiry	3.44	0.13
(3) Organization	1.20	1, 47	.280	.16	control	2.75	0.19
					inquiry	3.61	0.12
(4) Group Interaction	14.04	1, 47	<.001**	.48	control	3.09	0.14
					inquiry	3.60	0.12
(5) Individual Rapport	0.43	1, 47	.52	.10	control	2.94	0.18
					inquiry	3.83	0.10
(6) Breadth of Coverage	7.19	1, 47	.01	.36	control	2.92	0.16
					inquiry	3.52	0.13
(7) Examination	0.12	1, 47	.73	.05	control	2.54	0.16
					inquiry	3.52	0.10
(8) Assignments	0.01	1, 47	.91	.01	control	3.21	0.17
					inquiry	3.94	0.17
(9) Overall Instructional Ability	15.34	1, 47	<.001**	.50	control	2.63	0.19
					inquiry	3.59	0.09

* *p*< .0056, two-tailed. ** *p* < .0001, two-tailed.

### b) Cognitive Learning Evaluation Results

There was not a significant effect of GTA experience (in years) on the undergraduate responses to the CLE questions (see Table 4 for a list of CLE Factors) (*Pillai’s trace* = 0.08, *F*(6,40) = 0.60, *p* = .73, *partial η* ^2^ = 0.08, observed power = .21). There was a significant multivariate main effect for training group, (*Pillai’s trace* = 0.41, *F*(6,40) = 4.71, *p* < .001, *partial η* ^2^ = 0.41, observed power = .98; Table 11). However, there was not a significant effect for GTA academic program (*Pillai’s trace* = 0.03, *F*(6,40) = 0.17, *p* = .98, *partial η* ^2^ = 0.03, observed power = .09) or a significant interaction between training group and program (*Pillai’s trace* = 0.03, *F*(6,40) = 0.22, *p* = .97, *partial η* ^2^ = 0.03, observed power = .10). 

**Table 11:** MANCOVA test results for undergraduate Cognitive Learning Evaluation ratings (*n* = 50, α =.05).

**Effect / Interaction**	***Pillai's Trace***	***F***	***df***	***p***	***partial η^2^***	**power**
GTA Experience (in years teaching)	0.08	0.60	6, 40	.732	0.08	.21
Training Group	0.41	4.71	6, 40	.001*	0.41	.98
GTA Academic Program	0.03	0.17	6, 40	.984	0.03	.09
Training Group × GTA Academic Program	0.03	0.22	6, 40	.968	0.03	.10

* *p*<.05, two-tailed.

**Figure 2:**
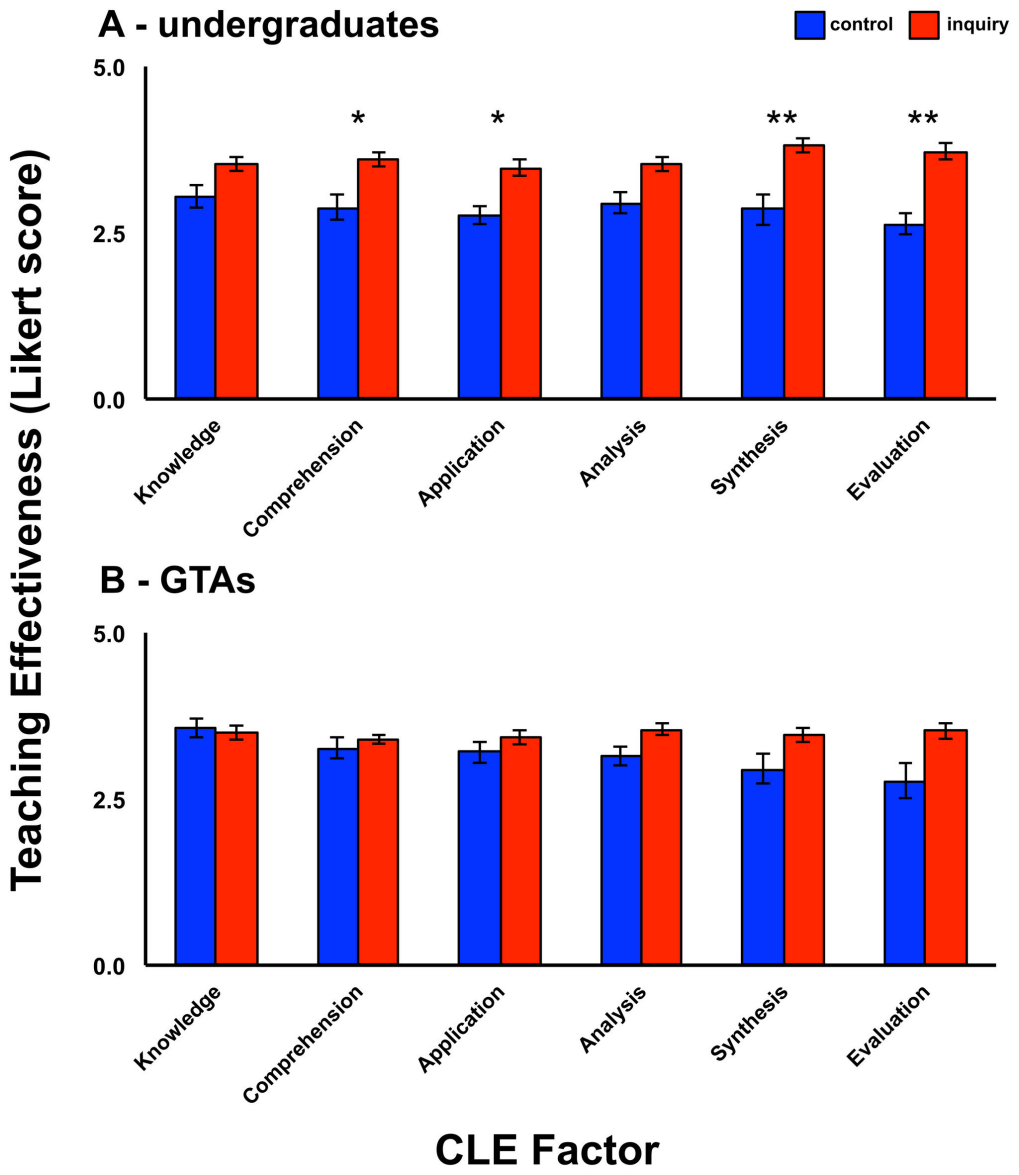
Mean undergraduate (A) and graduate teaching assistant (B) evaluations of GTA teaching effectiveness by Cognitive Learning Evaluation (CLE) questionnaire factor for the “best practices” (control) and inquiry-based learning pedagogy (inquiry) training groups (with standard error bars). Paired columns with stars are significantly different (Bonferroni-corrected significance levels: *p<0.0083, **p<0.001).

For the undergraduate responses, there was a significant univariate main effect for training group (using a Bonferroni-corrected α = .0083) for four of the six CLE factors, including: comprehension skills (CLE Factor 2), *F*(1,45) = 9.68, *p* < .003, *r* = .42; problem-solving/application skills (CLE Factor 3), *F*(1,45) = 9.76, *p* < .003, *r* = .42; synthesis skills (CLE Factor 5), *F*(1,45) = 11.22, *p* < .001, *r* = .48; and evaluation skills (CLE Factor 6), *F*(1,45) = 26.12, *p* < .0005, *r* = .61 (Figure 2, Table 12). A follow-up MANOVA revealed no difference in the significance level of any multivariate or univariate effects.

**Table 12:** ANCOVA test results for undergraduate Cognitive Learning Evaluation ratings (*n* = 50, α =.0083).

**CLE Factor**	***F***	***df***	***p***	***r***	**Training Group**	**Mean**	**SE**
(1) Knowledge	5.37	1, 45	.025	.33	control	3.04	0.16
					inquiry	3.54	0.11
(2) Comprehension	9.68	1, 45	<.001**	.42	control	2.88	0.18
					inquiry	3.61	0.12
(3) Application / Problem-solving	9.76	1, 45	<.001**	.42	control	2.77	0.14
					inquiry	3.47	0.13
(4) Analysis	7.68	1, 45	.008*	.38	control	2.95	0.17
					inquiry	3.52	0.10
(5) Synthesis	13.82	1, 45	<.001**	.48	control	2.85	0.23
					inquiry	3.82	0.12
(6) Evaluation	26.12	1, 45	<.001**	.61	control	2.63	0.17
					inquiry	3.72	0.12

* *p* < .0083, two-tailed. ** *p* < .0001, two-tailed.

There was not a significant effect of GTA experience (in years), on the GTA responses to the CLE questions (*Pillai’s trace* = 0.14, *F*(6,42) = 1.14, *p* = .36, *partial η* ^2^ = 0.14, observed power = .40; Table 13). There was not a significant multivariate main effect for training group, (*Pillai’s trace* = 0.19, *F*(6,42) = 1.68, *p* = .15, *partial η* ^2^ = 0.19, observed power = .57) or GTA academic program (*Pillai’s trace* = 0.10, *F*(6,42) = 0.79, *p* = .58, *partial η* ^2^ = 0.10, observed power = .28). In addition, there was not a significant interaction between training group and program (*Pillai’s trace* = 0.09, *F*(6,42) = 0.69, *p* = .66, *partial η* ^2^ = 0.09, observed power = .24). Given these results, univariate tests were not performed (Figure 2, Table 14). A follow-up MANOVA revealed no difference in the significance level of any multivariate or univariate effects.

**Table 13:** MANCOVA test results for GTA Cognitive Learning Evaluation ratings (*n* = 52, α = .05).

**Effect / Interaction**	***Pillai's Trace***	***F***	***df***	***p***	***partial η^2^***	**power**
GTA Experience (in years teaching)	0.14	1.14	6, 42	.359	0.14	.40
Training Group	0.19	1.68	6, 42	.149	0.19	.57
GTA Academic Program	0.10	0.79	6, 42	.583	0.10	.28
Training Group × GTA Academic Program	0.09	0.69	6, 42	.660	0.09	.24

*p<.05, two-tailed.

**Table 14:** ANCOVA test results for undergraduate Cognitive Learning Evaluation ratings (*n* = 52, α =.0083).

**CLE Factor**	***F***	***df***	***p***	***r***	**Training Group**	**Mean**	**SE**
(1) Knowledge	0.75	1, 51	.39	.12	control	3.57	0.14
					inquiry	3.49	0.10
(2) Comprehension	0.15	1, 51	.71	.05	control	3.27	0.78
					inquiry	3.40	0.42
(3) Application / Problem-solving	1.07	1, 51	.31	.14	control	3.20	0.14
					inquiry	3.42	0.12
(4) Analysis	5.98	1, 51	.02	.32	control	3.13	0.14
					inquiry	3.54	0.10
(5) Synthesis	4.41	1, 51	.04	.28	control	2.95	0.22
					inquiry	3.46	0.11
(6) Evaluation	6.45	1, 51	.01	.34	control	2.76	0.27
					inquiry	3.52	0.11

*p < .0083, two-tailed. ** *p* < .0001, two-tailed.

### c) Grades

**Figure 3:**
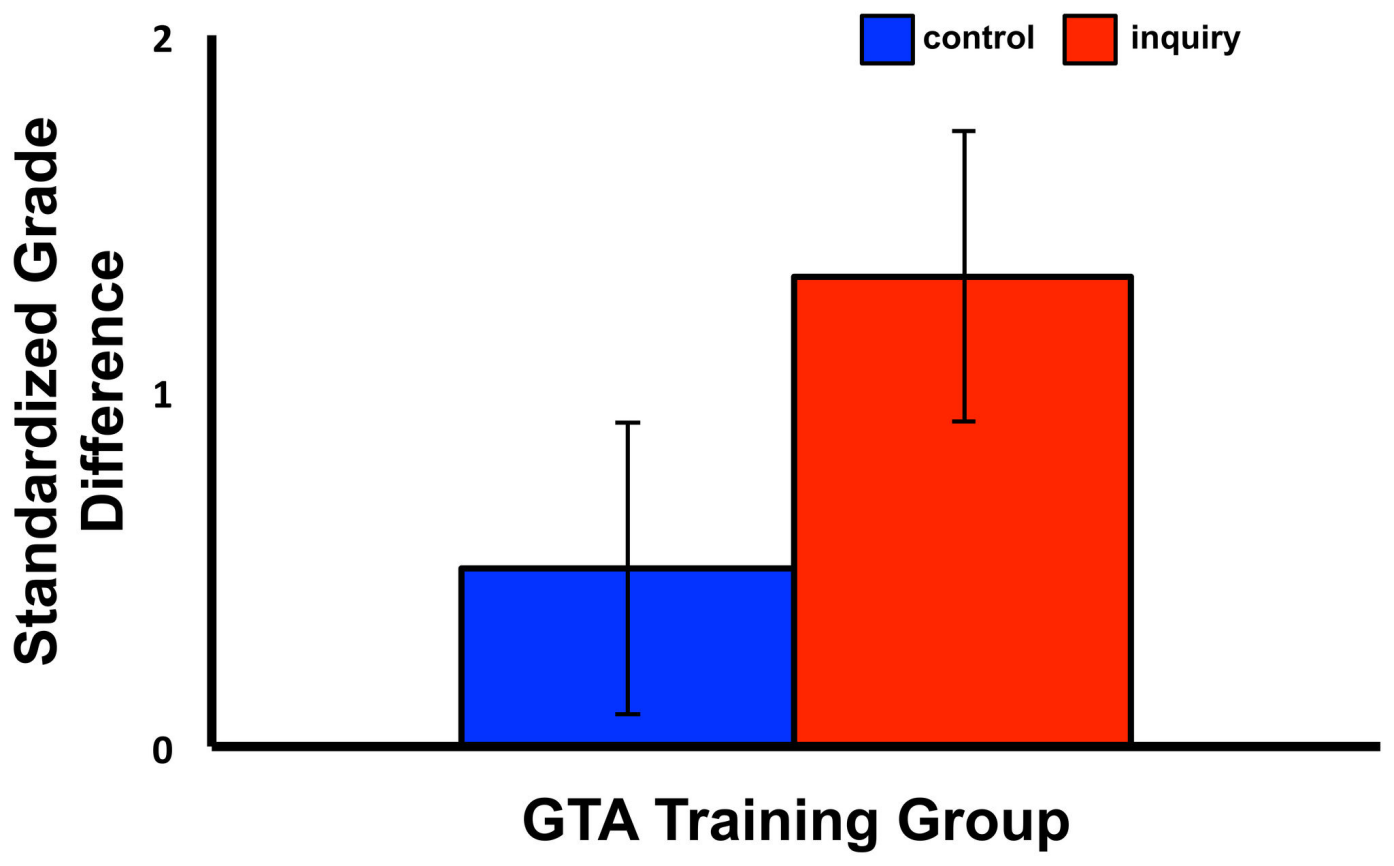
Mean standardized grade differences (in 12-point CGPA) for the “best practices” (control) and inquiry-based learning pedagogy (inquiry) training groups (with standard error bars).

There was a significant univariate main effect of training group on undergraduate grades in their biology courses, *F*(1,44) = 26.31, *p* < .022, *r* = .34, but not for program, *F*(1,44) = 1.66, *p* = .56, *r* = .19 ,or an interaction between training group and GTA academic program, *F*(1,44) = 0.53, *p* =.47, *r* = .11 (Figure 3, Table 15). 

**Table 15:** ANCOVA test results for standardized student grades (*n* = 49).

	***F***	***df***	***p***	***r***
GTA Experience (in years teaching)	0.72	1,44	.407	0.11
Training Group	0.90	1,44	.347	0.45
GTA Academic Program	12.29	1,44	.001*	0.12
Training Group × GTA Academic Program	0.05	1,44	.818	0.03

* *p*<.05, two-tailed.

## Discussion

These results suggest that offering inquiry-oriented pedagogy GTA training can yield gains in academic performance in undergraduate science labs and improve GTA teaching (as evaluated by both undergraduates and GTAs), particularly with respect to scientific reasoning. Inquiry pedagogy training group GTAs outperformed control training group GTAs, both with respect to SEEQ/CLE questionnaires and to self-reported undergraduate grades, and there were no items on either survey in which control training group GTAs received significantly higher ratings than inquiry pedagogy training group GTAs (this was true for both undergraduate evaluations and GTA self-evaluations). 

From the student- and self-evaluation data, GTAs who completed the inquiry pedagogy training received higher ratings than those in the control training group in two main areas: enthusiasm and interpersonal skills, and organization and academic value. This first result is fairly straightforward – the inquiry pedagogy training group was rated significantly higher than control training group GTAs for enthusiasm (SEEQ factor 2) and individual rapport (SEEQ factor 5), both of which are important skills in an interactive role such as a lab facilitator. The second result was that inquiry pedagogy training group GTAs received higher student ratings for organization (SEEQ factor 3) and assignments (SEEQ factor 7), both areas of the lab in which the GTA has only partial control, suggesting improvement in general teaching ability and planning skill. More specifically, undergraduates rated GTAs who completed the inquiry pedagogy training higher than those who completed the control training regimen for lower-order cognitive skills (e.g., SEEQ factor 1, CLE factors 2 and 3), higher-order cognitive skills (CLE factors 5 and 6) and overall measures of teaching ability (SEEQ factor 9). Taken together, these results indicate that GTAs who completed the inquiry pedagogy training were rated as better organized, provided better feedback on assignments, and were better overall teachers of both higher- and lower-order skills than the control training group GTAs. 

 We note that the differences between mean Likert scores for the two treatments are small in some cases. Likert scores, which have a limited range given that only five responses are possible (i.e. a score of 1-5), required consistently lower or higher values to register as significantly differing from the value of the other GTA training group. Since our analysis used a fairly conservative Bonferroni correction to decrease the likelihood of Type I errors, small but statistically significant differences between training groups for a specific SEEQ or CLE factor likely indicate improvements that are real but small. 

Our results indicated that self-reported grade data confirmed the advantage of inquiry pedagogy training, with the lab groups of GTAs who completed the inquiry-based learning pedagogy training reporting significantly higher standardized grades than GTAs who completed the control training regimen. These results suggest that undergraduate students in the lab sections of experimental group GTAs had higher overall course grades than did students in the lab sections of control training group GTAs. Because course grades include both a lab and a lecture component, higher overall grades may not be due entirely to influence of lab GTA, although it may be that effective lab teaching (from the GTA) may have additional in-lecture benefits that we did not quantify. Both training groups outperformed the average grade of the course, perhaps because some GTAs in the course did not have any training whatsoever. The finding that the grades of both GTA groups were higher than the course mean indicates that there might be some benefit to the control “best practice” training. The best practice training focused on practical skills and did use elements deemed successful in other studies (5). This type of GTA training might improve the academic value of lab activities for undergraduate students, but that GTAs, who are not expert teachers, may not be able to identify how or why.

We conclude that the recorded differences in (undergraduate- and GTA-rated) teaching ability between inquiry pedagogy training group GTAs and control training group GTAs are due to the fact that inquiry pedagogy GTA training improves GTAs’ ability to facilitate guided-inquiry activities related to scientific reasoning. While many methods can be used to teach undergraduates content, structured-inquiry activities (i.e. labs) are specifically designed to ask students to use higher-order cognitive skills as they employ the scientific method to solve problems. GTAs are evidently better able to teach undergraduates to “reason like scientists” after inquiry-based learning pedagogy training. We note that students rated inquiry pedagogy training group GTAs as better teachers of both the particular SEEQ factors related to inquiry facilitation (including academic value, enthusiasm, group interaction and rapport) as well as higher-order cognitive skills (i.e. higher CLE factors). We suggest that inquiry pedagogy training, which encourages GTAs to use active feedback as the lab activity is performed, has enabled inquiry GTAs to better teach using guided inquiry. 

 There are several limitations related to this study. Firstly, our sample size was small (52 GTAs), and the trial had only two training groups. Second, although there was a highly significant predictive relationship between training group and mean standardized grade for GTA lab section, lab grades were only part of the total grade; it is therefore unclear to what degree this treatment might predict improvement in student grades. Third, we note that we did not directly assess GTA teaching skills, and instead used student and GTA evaluations—along with self-reported student grades—to estimate actual GTA teaching ability. We also assume that the students who participated in our survey were representative of the larger undergraduate population. Further study, on a larger scale, of inquiry-based learning pedagogy training for science lab GTAs is our main recommendation for further studies. Although we believe our conclusions are supported by the data collected, a large, hierarchically-designed intervention experiment, involving a greater number of replicate training groups, would provide a more powerful test of our hypothesis. Further study of the reason of the differences between undergraduate and GTA questionnaire responses with respect to individual SEEQ and CLE factors might also be valuable; it is unclear why undergraduates seem better able to identify differences between training groups than the GTAs themselves.

## Conclusion

In conclusion, our data indicate that providing GTAs with a theoretical understanding of the guided-inquiry methods that underpin lab activities increases the quality of GTA teaching in introductory science labs, especially in areas related to scientific reasoning. Across all measures, inquiry-based learning pedagogical training for GTAs seems to improve teaching, even when training is limited to only five hours. We believe that our data indicate that there is value in including information on inquiry-oriented teaching methodologies in future GTA training regimens, especially where those GTAs teach inquiry-oriented activities such as teaching labs.
